# Transcriptomic profiling of the developing brain revealed cell-type and brain-region specificity in a mouse model of prenatal stress

**DOI:** 10.1186/s12864-023-09186-8

**Published:** 2023-02-24

**Authors:** Yuhao Dong, Jie Weng, Yueyan Zhu, Daijing Sun, Wei He, Qi Chen, Jin Cheng, Ying Zhu, Yan Jiang

**Affiliations:** Institutes of Brain Science, State Key Laboratory of Medical Neurobiology and MOE Frontiers Center for Brain Science, Fudan University, 200032 Shanghai, China

**Keywords:** Maternal stress, Embryonic brain, Gene transcription, Cell-type-specificity, Brain-region-specificity

## Abstract

**Background:**

Prenatal stress (PS) is considered as a risk factor for many mental disorders. PS-induced transcriptomic alterations may contribute to the functional dysregulation during brain development. Here, we used RNA-seq to explore changes of gene expression in the mouse fetal brain after prenatal exposure to chronic unpredictable mild stress (CUMS).

**Results:**

We compared the stressed brains to the controls and identified groups of significantly differentially expressed genes (DEGs). GO analysis on up-regulated DEGs revealed enrichment for the cell cycle pathways, while down-regulated DEGs were mostly enriched in the neuronal pathways related to synaptic transmission. We further performed cell-type enrichment analysis using published scRNA-seq data from the fetal mouse brain and revealed cell-type-specificity for up- and down-regulated DEGs, respectively. The up-regulated DEGs were highly enriched in the radial glia, while down-regulated DEGs were enriched in different types of neurons. Cell deconvolution analysis further showed altered cell fractions in the stressed brain, indicating accumulation of neuroblast and impaired neurogenesis. Moreover, we also observed distinct brain-region expression pattern when mapping DEGs onto the developing Allen brain atlas. The up-regulated DEGs were primarily enriched in the dorsal forebrain regions including the cortical plate and hippocampal formation. Surprisingly, down-regulated DEGs were found excluded from the cortical region, but highly expressed on various regions in the ventral forebrain, midbrain and hindbrain.

**Conclusion:**

Taken together, we provided an unbiased data source for transcriptomic alterations of the whole fetal brain after chronic PS, and reported differential cell-type and brain-region vulnerability of the developing brain in response to environmental insults during the pregnancy.

**Supplementary Information:**

The online version contains supplementary material available at 10.1186/s12864-023-09186-8.

## Background

Mental illness has become a significant global problem in the modern society and environmental stress is considered as one of the key risk factors [1]. Fetal brain is particularly sensitive to both internal and external insults during the long and complex development of the central nervous system (CNS), and prenatal stress (PS) is conceptually contribute to the neuropathogenesis of mental disorders through fetal programming [2]. Epidemiological studies reported that individuals with prenatal exposure to severe adverse life events had increased morbidity to neuropsychiatric dysfunctions, including intellectual disability, language impairment, conduct disorder, autism, schizophrenia, and mood disorders [3–5]. For example, retrospective studies on the 1998 North American ice storm reported that children with prenatal exposure had significantly lower score on intelligence and language ability compared to the control cohort [6, 7]. Animal studies have further validated that PS imposes negative influences on the structure and functional connectivity during brain development and increases susceptibility to stress in adulthood [8, 9]. Animals with PS experience demonstrated various neuropsychological behavioral abnormalities, including learning and memory deficits, social dysfunction [10], defensive withdrawal behavior [11], anxiety and depressive-like behaviors [12]. Therefore, PS-mediated adverse effects during early brain development may bear the neurobiological susceptibility for the long-term behavioral deficits on offspring in adulthood.

It has been reported that many factors mediate the harmful effects of PS on the fetal brain through the disturbance of the maternal neuroendocrine system [13]. One of the well-studied factors is the release of excessive glucocorticoids (GCs) caused by stress-induced activation of the hypothalamic-pituitary-adrenal (HPA) axis [14]. Under normal physiological conditions, the placental barrier protects the fetus from high glucocorticoids in pregnant women, primarily relying on the expression of 11beta-hydroxysteroid dehydrogenase type 2 (*11b-HSD-2*) in the placenta [15]. However, both human and animal studies have reported that chronic exposure to PS reduces the expression and activity of 11b-HSD-2 in the placenta and causes a sharp increase of GCs in the fetal brain [16–18]. Consequently, excessive GCs pose detrimental impacts on the development of critical brain regions [19, 20], which leads to the induction of oxidative stress and impairment of energy homeostasis [21–23]. GC receptor (GR) is encoded by *Nr3c1*, which is expressed in the fetal brain and down-regulated after adverse prenatal exposures [24–26]. GR belongs to the nuclear receptor superfamily of transcription factors, which can translocate into the nuclei upon the binding of GCs and affect many target genes, thus may contribute to the functional and structural impairments of multiple stress-sensitive brain regions [27, 28].

Besides the direct action of stress-induced steroid hormones, epigenetic changes also contribute to PS-induced transcriptional abnormalities of the fetal brain [13, 29−31]. Epigenetics refers to the regulation of gene expression without affecting DNA sequences, including many mechanisms like DNA methylation, histone modifications, non-coding RNAs, and chromatin remodeling [32]. Accumulating evidence indicated epigenetic dysregulations in both the fetal and adult brains of the offspring after the exposure of PS [13, 33−35]. For example, PS can induce changes of DNA methylation and histone modifications on promoters to alter gene transcription, such events were reported on *11b-HSD-2*[36, 37], brain-derived neurotrophic factor (*Bdnf*) and tropomyosin receptor kinase B (*TrkB*) [38], glutamic acid decarboxylase *Gad1* and *Gad2*[39], tryptophan hydroxylase 2 (*Tph2*) [40] and *Nr3c1* [25, 41]. Moreover, PS also disrupted the expression of DNA methylation enzymes (DNMT1 and TET1) [38] and histone deacetylases HDAC1 and HDAC2 [42], which may lead to the global disturbance of epigenetic landscape and thus cause genome-wide changes of gene expression. Indeed, besides the changes in the level of gene transcription, for example, genes involved in redox regulation [43], global changes of the general transcriptome were also reported in the fetal brain after PS [44–46]. Moreover, the reduction of O-linked N-acetylglucosamine transferase (OGT) altered the H3K27me3 signal in the placenta and mimicked the effect of PS, which caused a gender-specific transcriptomic disturbance in the fetal hypothalamus [47, 48]. Besides direct impacts on the fetal brain, transcriptomic alterations in adult brain after prenatal exposure of adverse events were reported in animal studies. For example, transcriptomic profiling on adult cortex [46, 49, 50], amygdala [51], and nucleus accumbens [50] in the animal model of maternal immune activation (MIA) identified many gene pathways critical for neurodevelopmental processes, including important genes associated with schizophrenia (SCZ), autism spectrum disorder (ASD) and cognitive phenotypes [52].

Despite many related studies conducted in this field, there is still a lack of systematic analysis of the transcriptome on the fetal brain after PS. Therefore, in the current study, we established a mouse model of PS model by applying chronic unpredictable mild stress (CUMS) during the pregnancy, and performed RNA-seq to examine gene transcription in the whole fetal brain at gestation day 15.5 (E15.5). We further performed cell-type and brain-region enrichment analysis using published single-cell RNA-seq (scRNA-seq) data and Allen in situ hybridization (ISH) data from the developing mouse brain. Our study provided an unbiased dataset for transcriptional alterations in the mouse brain after PS, and revealed distinct vulnerability of the fetal brain in response to stress in a cell-type and brain-region specific manner.

## Results

### Differential expression analysis

**Fig. 1:**
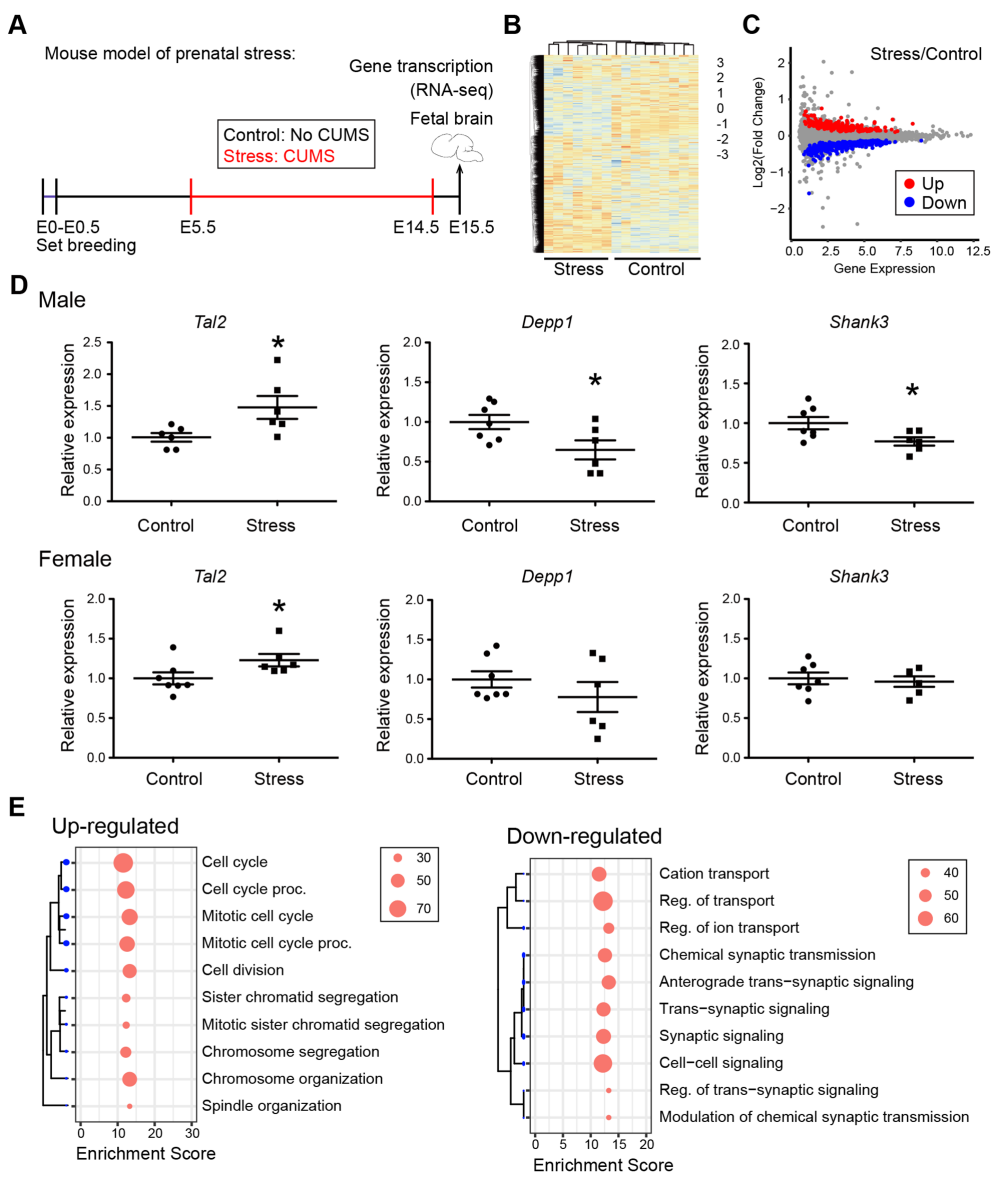
Transcriptomic profiling of the fetal brain after PS. (**A**) Mouse model of chronic prenatal stress. C57BL/6slac mice were used to set up timed pregnancy and plugged females were exposed to chronic unexpected mild stress (CUMS) between E5.5 and E14.5 (“Stress” group). Mice without CUMS exposure were used as controls (“Control” group). Whole fetal brains were collected at E15.5 for RNA-seq. (**B**) Clustered heatmap. N = 9 control/7 stress from 10 litters. (**C**) MA plot. Red, up-regulated genes; blue, down-regulated genes. Stress vs. Control, Padj < 0.05. (**D**) qRT-PCR validation in males and females, respectively. N = 6–7/group. Mean ± SEM. *P < 0.05, Unpaired t-test, one-tailed. (**E**) ShinyGO analysis (“Biological Process”) of up- and down-regulated genes. The size of dot indicates the number of genes in the enriched pathway. Enrichment score calculated as -Log10FDR.

In order to study the genome-wide transcriptional changes in the fetal brain after PS, we applied the pregnant female mice with CUMS during pregnancy between E5.5 and E14.5, and then performed RNA-seq using the whole brain (including forebrain, midbrain and hindbrain) harvested at E15.5 (Fig. 1A). Two separate batches with a total of 16 animals (7 “stress” and 9 “control”) from 10 litters were used and both sexes were included. Quality control analysis showed high sequencing coverage (on average 30 million paired reads per sample) and low technical variability across samples (Table S2). Clustering heatmap with normalized signal of all detected genes separated all samples by group (Fig. 1B). PCA plot displayed the separation of samples between the stress and control groups (PC1, 46%, PC2, 18%) (Figure S1A). Notably, there was clearly separation by sex among all the samples, which is in line with the literature that sex difference on the transcriptome level is present in the fetal brain, especially in response to stress or under neuropsychological conditions such as Alzheimer’s disease (AD), schizophrenia, drug abuse, depression, and anxiety [53]. We performed differential expression analysis (sex was included as a covariance) with all the samples and identified a total of 542 significant DEGs (*P*adj < 0.05), including 232 up-regulated and 310 down-regulated genes (Table S3A, Figure S1B). However, as shown on the MA-plot, the change of most significant DEGs was mild with the fold change less than 2-fold (stress vs. control, |Log_2_FC|<1) (Fig. 1C). We picked two DEGs with the largest fold changes: *Tal2* (up-regulated) and *Depp1* (down-regulated), and validated via real-time PCR in a different batch of male and female samples. *Tal2* was significantly increased in both males and females. *Depp1* was decreased in both sexes, but only reached significance in males (Fig. 1D). We, therefore, separated all the samples by sex and analyzed again. With the cutoff of *P*adj < 0.05, significant DEGs (46 up-regulated, 82-down-regulated) were only detected in males (Table S3C), but not in females. Alternatively, we used less stringent cutoff of *P* < 0.05 and detected 163 up-regulated, 276-down-regulated genes in females (Table S3E). Both sex-specific DEGs were largely overlapped with DEGs from the merged analysis (Figure S2A, D). We further validated *Shank3*, an important ASD (Autism spectrum disorder) risk gene that is critically involved in regulating synaptic plasticity, and found it was significantly decreased in the stressed males, but not in females (Fig. 1D).

Next, we performed gene ontology (GO) analysis of the overall DEGs with ShinyGO. We found the up-regulated genes were highly enriched in the pathways related to cell cycle (Fig. 1E, left panel), including many cyclin genes (*Ccna2*, *Ccnb1*, *Ccnb2*, *Ccnd2*, *Ccnf*, *Cdk1*, *Cdk4*) and cell-division genes (*Cdc20*, *Cdc42ep2, Cdca2*, *Cdca3*, *Cdca4*) (Table S3B). In line with it, Homer motif enrichment analysis on the promoters of these up-regulated DEGs identified several motifs for critical transcription factors involved in the regulation of cell-cycle-dependent genes (Figure S3A), including the nuclear factor Y (NFY) motif (CCAAT box) [54], the cell cycle genes homology region CHR promoter element (5’-TTTGAA-3’) [55], and the binding sites for E2F transcription factors [56]. Moreover, we found the down-regulated DEGs were highly enriched in the neuronal pathways, especially those related to the synaptic transmission (Fig. 1E, right panel), including several genes essential for synaptic structure and function, including *Syn1*, *Syn2*, *Syngr1*, *Syngr3*, *Syp*, *Syt17* (Table S3B). These genes can be differentially regulated in response to stress and are highly associated with various neuropsychological disorders [57–59]. Homer motif analysis on the promoters of the down-regulated DEGs identified significantly enriched motifs for NFkB, Zfp57, and Twist1 (Figure S3B). Recent study indicated that TWIST1 participated in dendritic remodeling and chronic stress-induced depressive behaviors [60]. Notably, “cell cycle” and “synaptic signaling” were the only two clusters of functional enriched pathways when GO analysis was performed using all the DEGs together (Figure S1C), and were also enriched for the DEGs from male-only and female-only analysis (Figure S2B, C, E, F, Table S3D, F).

### Gene-set enrichment analysis

**Fig. 2:**
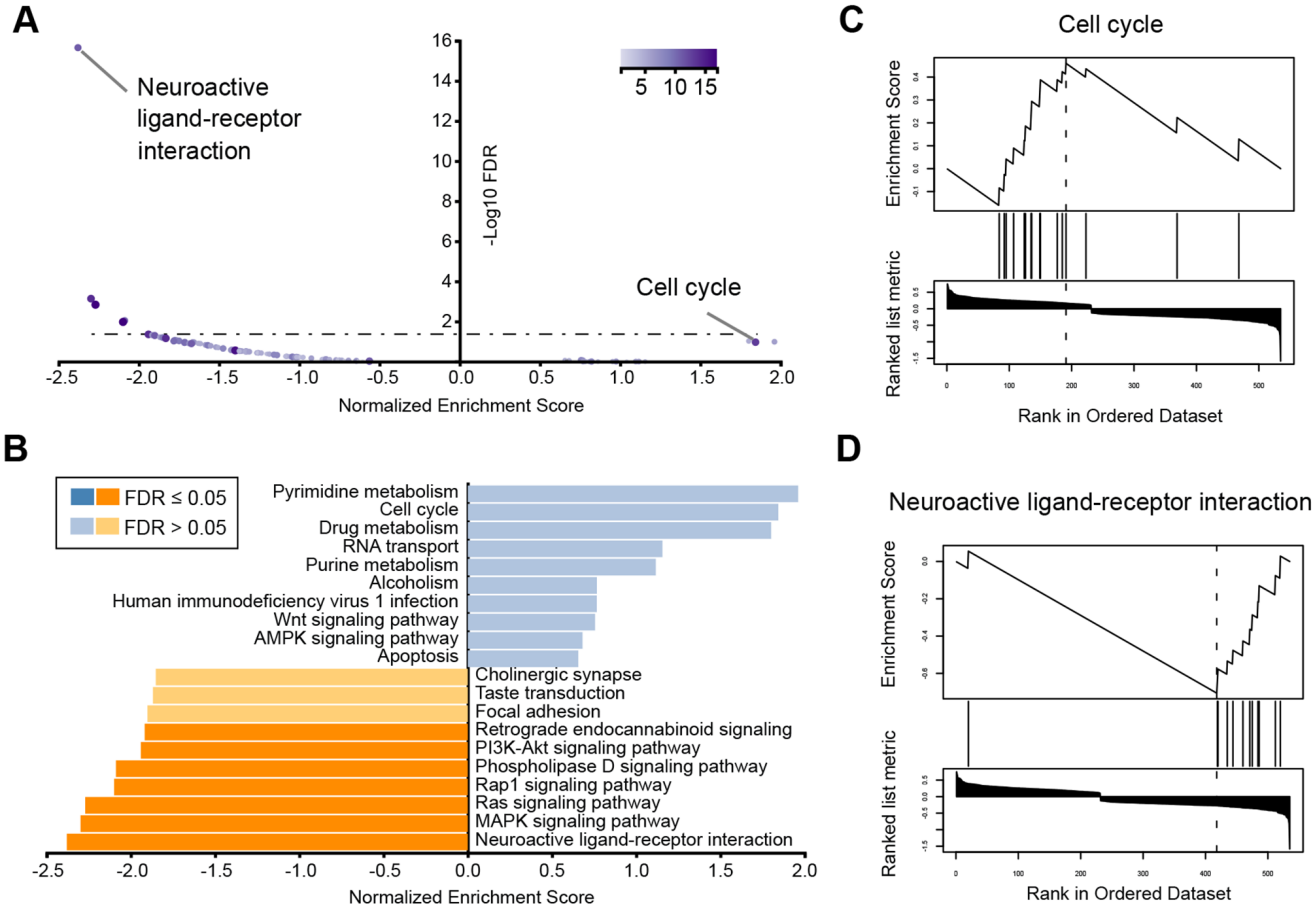
GSEA analysis of transcriptome in the fetal brain after PS. (**A**) Dot plot shows the pathway enrichment significance. Color key for number of genes in each pathway. (**B**) KEGG pathway enrichment (Stress vs. Control). Orange, gene sets positively associated with the Stress group. Blue, gene sets negatively associated with the Stress group. Top 10 pathways were shown. (**C**, **D**) The enrichment plots of “Cell cycle” (up-regulated) and “Neuroactive ligand-receptor interaction” (down-regulated) pathways

Alternatively, we performed the Gene Set Enrichment Analysis (GSEA) to identify differential KEGG pathways associated with the “stress” condition (Fig. 2, Table S4). We found in total 3 gene sets positively associated with the stress group (normalized Enrichment Score > 0, *P* < 0.05) (Fig. 2A). Consistent with abovementioned GO analysis on the DEGs, the “Cell cycle” pathway was highly enriched in the stress group, although it did not reach the significance of FDR < 0.05 (Fig. 2B, C), suggesting promoted cell proliferation in the fetal brain after PS. In addition, we found 22 gene sets negatively associated with the stress group (normalized Enrichment Score < 0, *P* < 0.05), and among them 7 gene sets reached the statistical significance (FDR < 0.05) (Fig. 2A). The top 10 pathways that were down-regulated in the stress group were critically involved in neuronal communication (Neuroactive ligand-receptor interaction, Retrograde endocannabinoid signaling, Cholinergic synapse) and cell signaling transduction (MAPK signaling, Ras signaling, Rap1 signaling, Phospholipase D signaling, PI3K-Akt signaling) (Fig. 2B, D). Accumulating evidence has linked PS experience with the impairment of synaptic plasticity and increased susceptibility of various mental disorders [61]. In the future study, it will be of interest to explore whether the observed dysregulation of gene expression in the fetal brain would contribute to the functional deficits in adulthood.

### Cell-type specific analysis

**Fig. 3:**
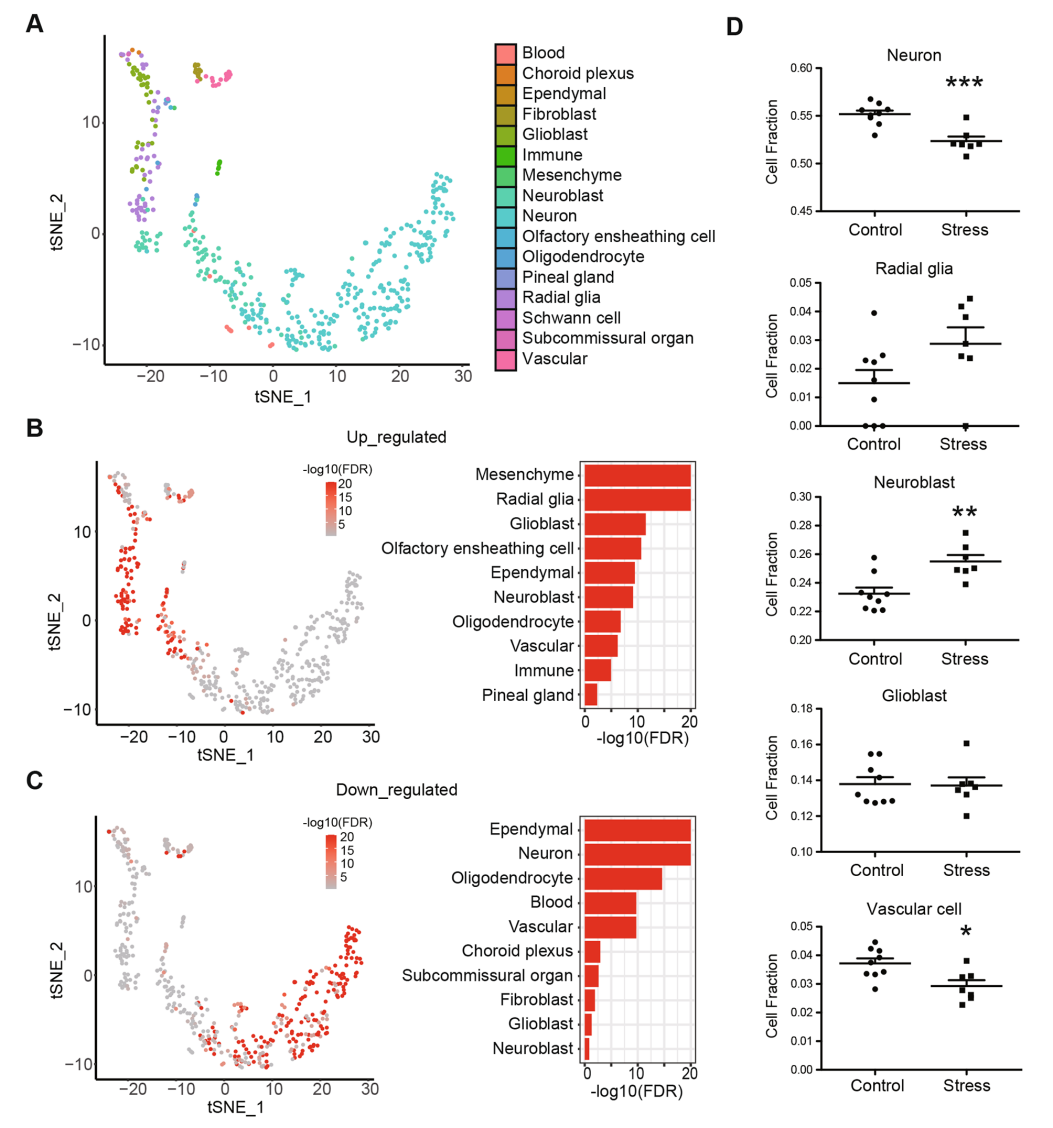
Integrative analysis of bulk RNA-seq and scRNA-seq. (**A-C**) Cell-type enrichment analysis of differentially expressed genes. (**A**) t-SNE plot shows cluster assignments of cells using published scRNA-seq data in mouse fetal brain at E15.5. (**B-C**) t-SNE plots and cell-type enrichment score for (**B**) up- and (**C**) down-regulated DEGs (FDR < 0.05). Notice up-regulated DEGs are highly enriched in the radial glia, while for down-regulated DEGs are enriched in the neurons. (**D**) Cell-type deconvolution analysis. Bar graphs show cell fractions (neuron, radial glia, neuroblast, gliobalst, vascular cell) in the control and stressed fetal brains. Notice significant decrease of neuron and decrease of neuroblast in the stressed brain. N = 9 control, 7 stress. Mean ± SEM. *P < 0.05, **P < 0.01, ***P < 0.001. Unpaired t-test

Neurogenesis is the key event that occurred during the early brain development, which consists of two major steps, proliferation of neural stem cells (also known as radial glia) and differentiation of postmitotic neurons. Interestingly, our data indicated that PS induced differential gene expression coupled with these two distinct processes, in which the up-regulated DEGs were highly associated with the regulation of cell proliferation, while the down-regulated DEGs involved in multiple critical functions of differentiated neurons (Figs. 1 and 2). We speculated that different type of cells in the fetal brain might be responsible for the differential effects. Therefore, we performed cell-type enrichment analysis using published scRNA-seq dataset [62] from the mouse fetal brain to extract the cell-type specific information from our current bulk RNA-seq data (Fig. 3, Table S5). This scRNA-seq dataset includes mass information for the whole fetal brain at multiple development time points [62]. We took out the data at E15.5 and identified 16 major classes of cells via unsupervised clustering analysis, covering the majority of known cell types in the fetal brain (Fig. 3A). We then applied the DEGs from the current bulk RNA-seq to this single-cell map, and performed cell-type enrichment analysis for the up- and down-regulated DEGs, respectively. In line with the functional enrichment analysis, we found the up-regulated DEGs were significantly enriched in the dividing cells including radial glia (neural stem cells) and mesenchyme stem cells, which can give rise to cells critical for brain-blood barrier integrity (Fig. 3B, Table S5A). Likewise, the down-regulated DEGs were primarily enriched in neurons and ependymal cells, which is considered as the 4th type of glia that line the ventricles of the brain (Fig. 3C, Table S5B). More detailed information about subclasses of cell types was including in the supplementary tables (Table S5C-D). Consistently, the up-regulated DEGs were highly enriched in all types of radial glia, as well as neuroblast, glioblast and angioblast, which are different types of precursors in CNS. Immune cells like cycling microglia was also on the top of the list for the down-regulated DEGs. The down-regulated DEGs, as expected, were enriched in multiple type of neurons from mixed regions of brain, including Cajal-Retzius, GABAergic, glutamatergic, glycinergic, serotonergic and dopaminergic neurons. Together, this implied the unique cell-type specificity for differentially transcriptional regulation in the fetal brain in response to PS.

Gene transcription is critically involved in the cell-fate decision during brain development. Therefore, transcriptional alterations of cell-type specific genes may also influence the cell composition in the fetal brain in response to PS. For example, the observed up-regulation of cell-cycle genes may increase cell proliferation and thus may increase the absolute number of dividing cells in the stressed brains. To check whether cell composition was altered in response to PS, we performed cell deconvolution analysis of our bulk RNA-seq using the same set of scRNA-seq data. As showed in Table S6, the major cell populations in the fetal brain at E15.5 are neurons (53.94%), neuroblasts (24.23%) and glioblasts (13.75%) (Figure S4A), which reflects the peak of neurogenesis at this stage. Notably, as compared to the control, there was a subtle but very significant decrease (*P* < 0.001) of the fraction of neuron in the stressed group (Fig. 3D). Different from differentiated neurons, the alterations in dividing cells were more complex. On average, radial glia only account for 2.1% in our dataset, and there was a trend (*P* = 0.0783) of increase in the stress group (Fig. 3D). Radial glia are neural stem cells that can give rise to both neuroblasts and glioblasts, which further generate neurons and glia, respectively. Interestingly, while the fraction of neuroblast was significantly increased (*P* < 0.01) in the stressed group, there was no significant change for glioblast (Fig. 3D). Moreover, the fraction of vascular cell, which includes angioblast, pericyte and endothelial cell, was significantly decreased (*P* < 0.05) (Fig. 3D). No significant change was observed for olfactory ensheathing cell or blood cell (Figure S4B). Overall, this implied compromised neurogenesis in the stressed fetal brain with the arrestment at the stage of neural precursors, which failed to commit cell differentiation towards neuronal lineage.

### Brain-region mapping of DEGs

**Fig. 4:**
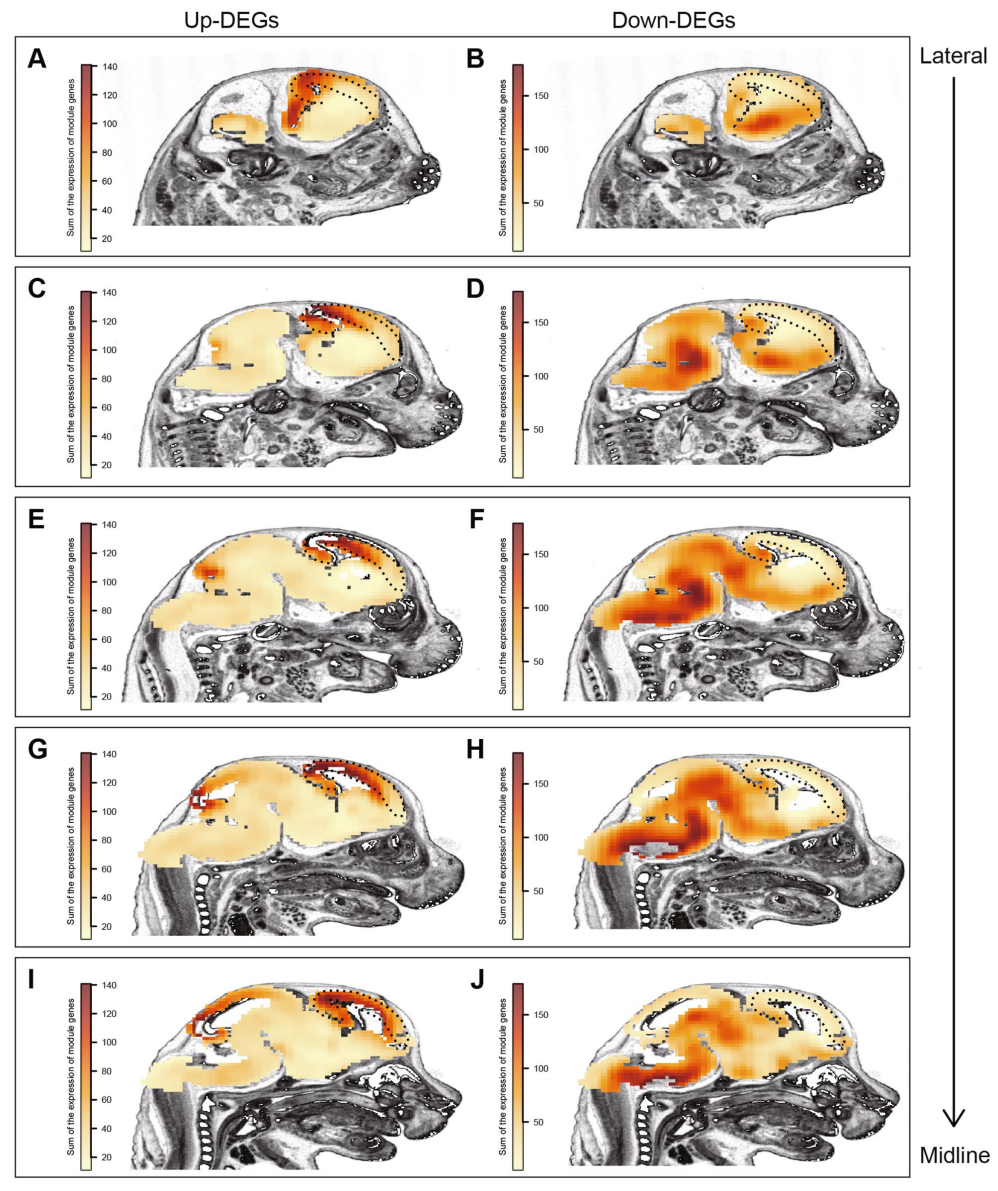
Brain region mapping for differential expressed genes in response to PS. Visualization of the expression pattern for (**A, C, E, G, I**) up- and (**B, D, F, H, J**) down-regulated genes on the Allen developing mouse brain at E15.5 (sagittal section from lateral to midline). Dashed-line circles cortical plate and hippocampal formation. Notice distinct expression pattern between up- and down-regulated DEGs, in which up-regulated genes are primarily enriched in the dorsal forebrain, while down-regulated genes in the ventral mid- and hind-brains

Brain is one of the most vulnerable organs under stress and some brain regions in the forebrain have been heavily studied in previous studies, including prefrontal cortex (PFC), hippocampus, and amygdala [63–66]. Meanwhile, other brain regions are also critically involved in the response to stress, for example, the ventral tegmental area (VTA) in the midbrain [67] and the nucleus of the solitary tract (NTS, solitary nucleus) in the dorsomedial medulla [68]. In our current study, we used the whole fetal brain to study the transcriptomic alteration after PS. To extract the potential brain-region specific information, we mapped the DEGs onto the Allen developing mouse brain atlas (sagittal sections, from lateral to midline) (Figure S5) and visualized the expression pattern using the available ISH data at E15.5. Interestingly, the brain-region expression pattern was remarkably different between the up- and down-regulated DEGs (Fig. 4). The signals of up-regulated DEGs were primarily enriched in the dorsal part of the forebrain, including cortical plate and hippocampal formation (Fig. 4A, C, E, G, I). This is consistent with the finding that up-regulated DEGs were enriched in the radial glia and the cell-cycle pathways, as neurogenesis is particularly active in neocortex at this developmental stage. Surprisingly, there was relatively low signal of the down-regulated DEGs in the cortical plate. Rather, these genes were preferentially enriched in the ventral part of the fetal brain, especially in the midbrain and hindbrain (Fig. 4B, D, F, H, J). This might be explained by the fact that brain regions in the mid-and hind-brain develop much earlier as compared to the forebrain, therefore, most of the cells in these regions already exit the cell-cycle and undergo differentiation at E15.5. This is also in line with the cell-type and functional enrichment for the down-regulated DEGs, which were primarily affecting cell communication and signaling transduction in neurons.

## Discussion

In the current study, we first established a mouse model of PS in which the pregnant females were exposed to CUMS from E5.5 to E14.5, the most critical period of neurogenesis in the early brain development. We then collected the whole brains from the offspring at E15.5 and performed RNA-seq to analyze the transcriptomic difference between the stressed brains and controls. Genome-wide alterations on the gene expression were observed in the stressed brain as compared to the controls, although the fold change of individual gene was relatively small. GO analysis revealed distinct enriched functional pathways for up- and down-regulated DEGs, respectively. The up-regulated DEGs were primarily enriched in the cell cycle pathways, while the down-regulated DEGs were significantly enriched in multiple pathways related to neuronal functions, especially the synaptic communication. Next, we performed cell-type enrichment analysis using published scRNA-seq of the mouse fetal brain, and found that the up-regulated DEGs showed high enrichment in radial glia, while the down-regulated DEGs enriched in different type of neurons. Cell deconvolution analysis also showed alterations of cell fraction in the stressed brain, indicating accumulation of neuroblast and impaired transition to the neuronal lineage. Moreover, spatial mapping on the developing Allen brain atlas revealed distinct expression pattern for up-and down-regulated DEGs. The up-regulated DEGs were mainly expressed in the dorsal forebrain including the cortical plate and hippocampus. Surprisingly, the signal of down-regulated DEGs were largely located on the ventral part of the fetal brain, especially the midbrain and hindbrain. Taken together, our study provided an unbiased transcriptomic data source and revealed high cell-type- and brain-region-specificity in the fetal brain in response to PS.

The detrimental effect on the brain after excessive exposure to GC is primarily mediated by its receptor GR, which is best studied in the model of maternal care [69, 70]. The promoter of *Nr3c1* was hypermethylated and its expression was down-regulated in the hippocampus of offspring with a lack of good maternal care, which blunted the negative feedback regulation of the HPA-axis and contributed to a decreased resilience to stress in adulthood [70]. In our current study, down-regulation of *Nr3c1* was also observed in the stressed fetal brain, although the change was very subtle (Log_2_FC = -0.21, *P* = 0.0085, Stress vs. Control, all animals). We checked *Nr3c1* in the mouse developing brain on Allen brain atlas and found its expression is only present in a limited number of cells at E15.5, particularly in the midbrain and hindbrain. Therefore, GC might specifically affect specific types of neurons in specific brain regions at the embryonic stage, and such effect was diluted when we used the whole brain as starting material. On the other hand, this is in line with our data showing the down-regulated genes that are essential for neuronal functions were highly enriched in the midbrain and hindbrain. It will be interesting to identify these *Nr3c1*/GR positive cells and figure out whether these are the critical cells directly mediating the negative effect of PS on the fetal brain.

Numerous studies using animal models of PS reported neurodevelopmental impairments and long-lasting deficits in adulthood [4, 5, 71, 72]. For example, it has been reported that multiple aspects of neurodevelopment were compromised in a rat model of PS, including neurogenesis, migration, differentiation and synapse refinement, accompanied by long-term changes in the dopamine sensitivity of offspring in adulthood [73]. Consistent with the published literatures, both differential expression analysis and GSEA of our current RNA-seq data suggested the PS-induced dysregulation of genes in the fetal brain, which are essential for neurodevelopment. Notably, many genes in the cell cycle pathway were up-regulated in the stressed brain, while genes implicated for the neuronal communication were down-regulated. We further extract cell-type information out of our bulk RNA-seq using published scRNA-seq data and found out that indeed transcriptional up-regulation were primarily occurred in those dividing radial glia cells, while down-regulation was associated with all types of neurons, including glutamatergic, GABAergic, dopaminergic, serotoninergic and glycinergic neurons. Together, it suggested that PS may impair neurogenesis by promoting proliferation of neural stem cells, but in general, impose adverse impacts on mature neurons. This was further supported by the results from cell deconvolution analysis, which showed the selective increase of neuroblasts and decrease of neurons. It is worthwhile to mention that most events for neuronal communication, especially synaptic transmission, has not yet occurred in the fetal brain at E15.5. Therefore, follow-up study will continue to address the question whether the observed decrease of neuronal gene expression may contribute to the PS-induced long-term neurological impairments in adulthood.

One interesting finding from our current study is the brain-region-specificity for the DEGs in the stressed brain. In the literature, most studies were focused on forebrain regions, including the cortex, hippocampus, and amygdala [74]. Potential reasons are: (1) preclinical and clinical studies on PS observed structure changes on these brain regions; (2) these brain regions are critical for emotion and cognitive functions, which are compromised in the individuals with PS experience. And indeed, in our current study, up-regulated genes were shown to be enriched in the cortical plate and hippocampal formation. However, surprisingly, the down-regulated genes were most enriched in ventral part of the fetal brain, especially some regions in the midbrain and hindbrain. There are some supporting evidences in the literature. Studies on the midbrain reported the serotonergic and dopaminergic neurotransmission deficits after PS [75, 76]. Moreover, epigenetic alterations in the developing brain were reported to mediate mood behavioral changes in a mouse model of postnatal early life stress, and the molecular changes were much more robust in certain cell populations, including medium spine neurons in nucleus accumbent [77–79]. Hindbrain coordinates important functions that are fundamental to body processes. Recent studies also suggest its role in response to stress, mediated by the glucagon-like peptide-1 (GLP-1) neurons in the nucleus of the solitary tract (NTS) in the dorsomedial hindbrain [68, 80]. In the future work, integrative analysis between scRNA-seq, spatial transcriptomics and live-cell labeling will offer more precise mapping for cell-type-specific and brain-region-specific changes in the CNS after the exposure to PS.

There are many questions to be addressed in the current study. Sex bias is a commonly considered factor in stress-related studies. Both sexes were included in the current study. Although only males showed significant changes (FDR < 0.05) on the transcriptome, the females showed the same trend with a less stringent cutoff (*P* < 0.05) and affected similar functional pathways. Therefore, the observed global sex difference in our current study might be due to small-fold changes in individual genes and large variability among samples. Still, our data suggested that the fetal brains of the male mice were more sensitive to PS as compared to the females. Besides, our current study was performed on the whole fetal brain boosted by the bioinformatic analysis using published scRNA-seq and brain-region expression information from Allen Brain Atlas. Future studies focusing on selective brain regions or cell types will be needed to provide validation and allow more detailed mechanistic studies. Despite all these challenges, our current study using unbiased transcriptomic profiling indicated cell-type and brain-region specific changes of gene expression in the fetal brain after PS, which provide insight for future study deciphering the molecular mechanisms underlying PS-mediated negative effects on the brain and behaviors.

## Conclusion

In summary, this study provided an unbiased data source for transcriptomic alterations of the whole fetal brain after chronic PS, and revealed a potential pattern of PS transcriptomic alternations reporting differential cell-type and brain-region vulnerability of the developing brain in response to environmental insults during the pregnancy.

## Methods

### Animals

All C57BL/6Slac mice were purchased from Slake Laboratory Animal Co., Ltd, Shanghai, China, and housed in per independent cage at room temperature, with a 12–12 h day/night cycle. All mice were allowed sterile water and food ad libitum. All animal work was approved by the Animal Care and Use Committee of Shanghai Medical College of Fudan University.

### Mouse model of prenatal stress

Adult mice were used to set up timed pregnancy and subjected to CUMS. In brief, female and male mice of similar age were caged together at 5:00–6:00pm and defined as E0. The next morning, the vaginal plug was checked at 9am (E0.5), and female mice with plug were separated and individually housed. All the plugged females were then divided into “stress” and “control” groups. For the stress group, mice were exposed to CUMS according to the published protocols with some modifications [81–83], see Table S1 for details. To minimize abortion risk during the first trimester, CUMS exposure started at E5.5 and lasted for 10 days until E14.5. Meanwhile, animals in the control group remained in the home cages. At E15.5, all pregnant mice were sacrificed, litters collected, whole fetal brain tissues (including forebrain, midbrain and hindbrain) harvested, freshly frozen, and stored at -80 °C for subsequent RNA-seq analysis. Two batches of 16 animals from 10 litters were used, both sexes included (Table S2).

### RNA-seq

Total RNA was extracted from the fresh frozen E15.5 fetal brain samples using the RNeasy Lipid Tissue Mini Kit (Qiagen Cat#74,804). Briefly, brain tissue was homogenized in 500 µl of Trizol reagent and mixed thoroughly with 100 µl of chloroform, centrifuge at 12,000 g at 4 °C for 15 min. The upper aqueous phase was transferred and mixed with an equal volume of 70% ethanol, and then transferred to RNeasy Mini spin column followed by RW1 wash, DNaseI treatment, RPE wash twice, and ddH20 elution. Final RNA concentration was measured and then sent to BGI Genomics, China, for mRNA library preparation and deep sequencing with paired-end, 100 bp (PE100).

### Real-time RT-PCR

Total RNA was extracted from E15.5 brain tissues from different batch of mice, reverse-transcribed using iScript™ cDNA Synthesis Kit (Bio-Rad, 1,708,891), and PCR amplified using Power SYBR™ Green PCR Master Mix (Thermo, 4,368,702) on Thermo Fisher Scientific Applied Biosystems QuantStudio5. Primers used were: *Tal2* (forward, CAGCTACCTTGACTGCGC, reverse, GTCTCGTTCTTGCTCAGCTT); *Depp1* (forward, CACATCGTCCTGACTGTCCT, reverse, TCCCGAATCGTTGGCAAATG); *Shank3* (forward, GACACTGAGGCCGGACATT, reverse, CCCCTACAGATTTGGTCCGT); *Gapdh* (forward, GTCTTCTGGGTGGCAGTGAT, reverse, GGTCCTCAGTGTAGCCCAAG). *Gapdh* was used as the reference gene.

### Data Analysis

#### RNA-seq

FastQC (Babraham Bioinformatics - FastQC A Quality Control tool for High Throughput Sequence Data) was first used for quality controls including the sequence quality, GC and N contents, length distribution, and duplication levels. Trim-galore (Babraham Bioinformatics - Trim Galore!) was then used to remove short and low-quality reads and adapters (--quality 20 --phred33 --stringency 1 --length 20 --paired). Paired-end clean reads were aligned to the reference genome (UCSC, mm10) using Tophat2 v2.1.1 [84] (--max-multihits 20, --transcriptome-max-hits 60; --GTF gencode.vM20.annotation.gtf). Samtools v1.9 [85 was used to sort and build the alignment files index. FeatureCounts v1.6.3 [86] from the subread package was used to get raw counts mapped to gene exons (-p -t exon -g gene_id; -a gencode.vM20.annotation.gtf).

Differential analysis was generated by using DESeq2 (DOI: 10.18129/B9.bioc.DESeq2. DESeqDataSetFromMatrix constructs the DESeq data set, estimateSizeFactors calculates the sample size factors to normalize the count matrix, estimateDispersons evaluates and corrects the dispersion, nbinomWaldTest performs statistical tests, and finally extracts the differential analysis results with ‘independentFiltering = FALSE’). Principal component analysis (PCA) and heatmap were generated with regularized logarithm (rlog) transformed values from DESeq2 and batch effect was removed by Combat function [87] from sva r package, which provides a correction model based on an empirical Bayesian framework (ComBat(data = data, batch = batch, mod = mod)). Plots was drawn by ggplot2 and pheatmap r packages. In the differential algorithm, sex and batch variables were both added in the design formula, while results were generated with stress condition as the variable of interest (DESeqDataSetFromMatrix(countData, colData = colData, design = ~ sex + batch + condition)). *Padj* < 0.05 was set as the threshold for the significantly differential expression.

Functional enrichment analysis on the differential expressed genes was performed by using ShinyGO v0.61 [88] (GO Biological Process, *P*-value cutoff (FDR) = 0.05; http://bioinformatics.sdstate.edu/go/). Gene Set Enrichment Analysis (GSEA) was performed with WebGestalt (WEB-based Gene SeT AnaLysis Toolkit) [89] using KEGG functional database [90] and all DEGs were ranked by log2FoldChanges.

#### Integrative analysis of bulk RNA-seq and scRNA-seq

Cell-type enrichment analysis was generated with differentially expressed genes (DEGs) from our bulk RNA-seq data and the published scRNA-seq data of E15.5 mouse brain [62] (http://mousebrain.org/downloads.html). Cell types were clustered as classes and subclasses. First, cell type expression was indicated with means from single cells, and the whole expression matrix was normalized into PEM matrix to adjust the sample bias:

$$\begin{array}{l}PEM = lo{g_{10}}\frac{{Xi}}{{\sum\nolimits_{i = 1}^n {Xi*\frac{{Si}}{{\sum\nolimits_{i = 1}^n {Si} }}} }} = \\{\log _{10}}\left( {\frac{{\sum\nolimits_{i = 1}^n {Si} }}{{Si}}*\frac{{Xi}}{{\sum\nolimits_{i = 1}^n {Xi} }}} \right) = \\{\log _{10}}EEi\end{array}$$

Si summary of the expression of all genes in cell type i. And Kolmogorov–Smirnov test (K-S test) was then performed in each cell type of scRNA-seq data, between DEGs and other unchanged genes (*P*adj > 0.05) from current bulk RNA-seq data to assess enrichment based on the distribution of the gene sets. *P* value was adjusted with BH method. In the results, *P-*value refers to the enrichment of DEGs for different cell types from the single-cell RNA-seq dataset. The significance of the *P*-value indicates the difference of expression level between DEGs and non-DEGs in certain cell type.

Cell-type deconvolution analysis of our bulk RNA-seq was calculated by CIBERSORTx [91]. Expression matrices of all genes from our bulk RNA-seq data and the published scRNA-seq data were generated, and the Cell-Fractions module from CIBERSORTx was used to perform an estimation of the proportions of distinct cell clusters in the bulk RNA-seq.

#### Brain region mapping of differential expressed genes

R package brainmapr [92] (https://github.com/hms-dbmi/brainmapr) was used to explore the spatial expression patterns of modules in the developing mouse brain at E15.5. The package provided visualization of the in situ hybridization (ISH) gene expression data from the Allen Developing Mouse Brain Atlas. Sagittal sections were used from E15.5 reference atlases (Image Thumbnails :: Allen Brain Atlas: Developing Mouse Brain (brain-map.org)). For a certain module, its spatial expression level was calculated as the sum of the expression values of the ISH data of the available genes in the module.

## Electronic supplementary material

Below is the link to the electronic supplementary material.

**Additional file 1. Figure S1. **Transcriptomic profiling of the fetal brain after PS.

**Additional file 2. Figure S2.** Transcriptomic profiling of the fetal brain after PS in males and females.

**Additional file 3. Figure S3.** Motif analysis of differentially expressed genes in the fetal brain after PS.

**Additional file 4. Figure S4.** Cell deconvolution analysis.

**Additional file 5. Figure S5.** Schematic diagram shows sagittal sections picked from lateral to midline for Figure 4.

**Additional file 6. Table S1.** Protocol for mouse model of prenatal stress.

**Additional file 7. Table S2.** RNA-seq QC, N=9 control, 7 stress.

**Additional file 8. Table S3A. **Differential genes list of RNA-seq, Stress v.s. Control. Padj < 0.05; N=9 control, 7 stress**. Table S3B. **GO analysis of DEGs, Stress v.s. Control. Padj < 0.05; N=9 control, 7 stress. **Table S3C.** Differential genes list of male samples, Stress v.s. Control. Padj < 0.05; N=5 control, 3 stress. **Table S3D. **GO analysis of DEGs from male samples, Stress v.s. Control. Padj < 0.05; N=5 control, 3 stress. **Table S3E.** Differential genes list of female samples, Stress v.s. Control. Pvalue < 0.05; N=4 control, 4 stress. **Table S3F.** GO analysis of DEGs from female samples, Stress v.s. Control. Pvalue < 0.05; N=4 control, 4 stress.

**Additional file 9. Table S4A.** Top 10 pathways in positive and negative GSEA results of all differential expressed genes, Stress v.s. Control. N=9 control, 7 stress.

**Additional file 10. Table S5A.** Cell type specific enrichment analysis of Class with up-regulated genes. Log2FC > 0; Padj < 0.05. N=9 control, 7 stress. **Table S5B.** Cell type specific enrichment analysis of Class with down-regulated genes. Log2FC < 0; Padj < 0.05. N=9 control, 7 stress. **Table S5C.** Cell type specific enrichment analysis of SubClass with up-regulated genes. Log2FC > 0; Padj < 0.05. N=9 control, 7 stress. **Table S5D**. Cell type specific enrichment analysis of SubClass with down-regulated genes. Log2FC < 0; Padj < 0.05. N=9 control, 7 stress.

**Additional file 11. Table S6.** Cell deconvolution analysis of current bulk RNA-seq using published scRNA-seq dataset from the mouse fetal brain at E15.5.

## Data Availability

(ADM) All the raw and processed deep sequencing data were submit to GEO (GSE205087).
