# Supplementary material for: Transcriptomic profiling of the developing brain revealed cell-type and brain-region specificity in a mouse model of prenatal stress
Source: BMC Genomics. 2023 Feb 24;24:86. doi: 10.1186/s12864-023-09186-8 (PMC9951484; doi:10.1186/s12864-023-09186-8)
Supplement: Supplementary file 1 — Additional file 1. Figure S1. Transcriptomic profiling of the fetal brain after PS. [file 12864_2023_9186_MOESM1_ESM.pdf]

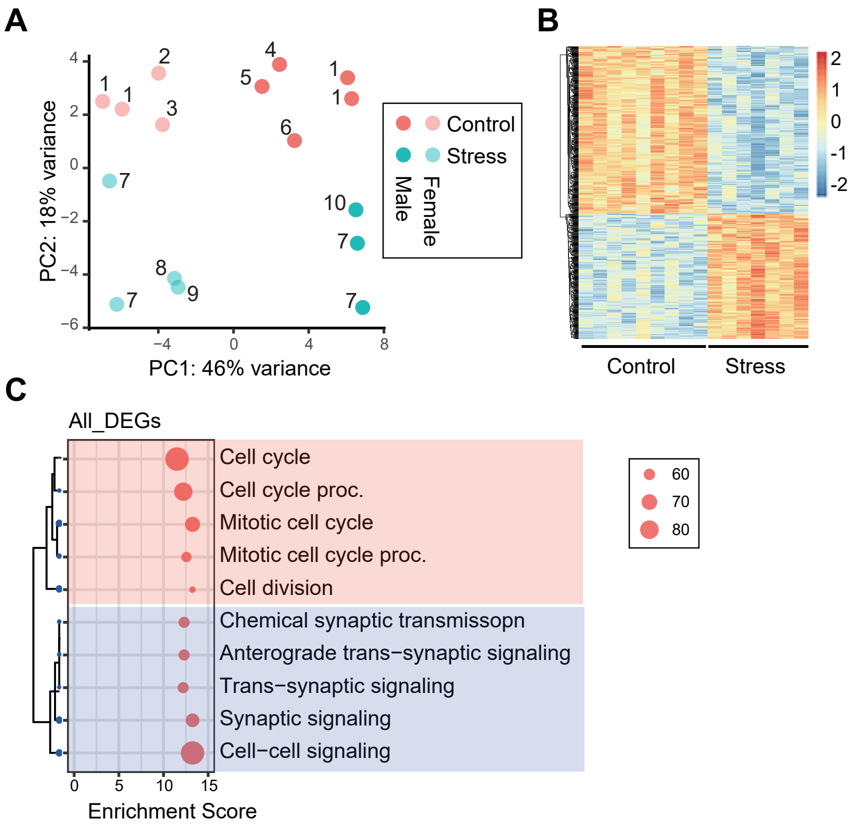

Figure S1: Transcriptomic profiling of the fetal brain after PS. (**A**) PCA plot for 9 "control" and 7 "stress" samples from 10 litters including both sexes. (**B**) Heatmap shows differential gene expression in the control and stress groups. (**C**) ShinyGO analysis ('Biological Process') of all DEGs. The size of dot indicates the number of genes in the enriched pathway. Enrichment score calculated as -Log_{10}FDR.
