# Supplementary material for: Transcriptomic profiling of the developing brain revealed cell-type and brain-region specificity in a mouse model of prenatal stress
Source: BMC Genomics. 2023 Feb 24;24:86. doi: 10.1186/s12864-023-09186-8 (PMC9951484; doi:10.1186/s12864-023-09186-8)
Supplement: Supplementary file 2 — Additional file 2. Figure S2. Transcriptomic profiling of the fetal brain after PS in males and females. [file 12864_2023_9186_MOESM2_ESM.pdf]

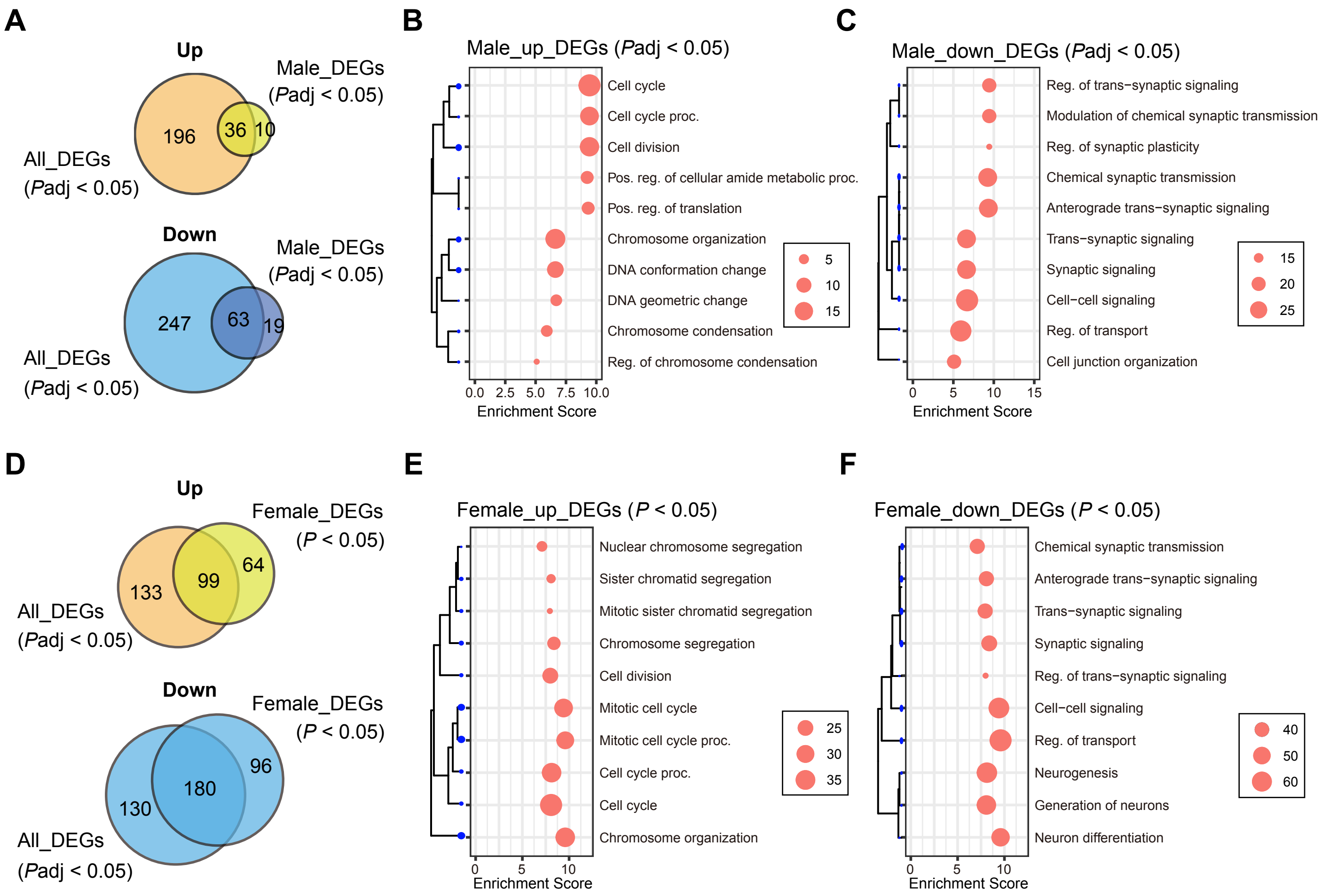

Figure S2: Transcriptomic profiling of the fetal brain after PS in males and females. (**A**) Venn plot shows overlap between DGEs from all samples and males only (Stress vs. Control). *P*adj < 0.05, N=9 control/7 stress (all); *P*adj < 0.05, 5 control/3 stress (male). (**B, C**) ShinyGO analysis (‘Biological Process’) of DEGs in males (*P*adj < 0.05). The size of dot indicates the number of genes in the enriched pathway. Enrichment score calculated as -Log_{10}FDR. (**D**) Venn plot shows overlap between DGEs from all samples and regulated genes from females only (Stress vs. Control). *P*adj < 0.05, N=9 control/7 stress (all); *P* < 0.05, 4 control/4 stress (female). (**E, F**) ShinyGO analysis (‘Biological Process’) of regulated genes in females (*P* < 0.05).
