# Supplementary material for: Transcriptomic profiling of the developing brain revealed cell-type and brain-region specificity in a mouse model of prenatal stress
Source: BMC Genomics. 2023 Feb 24;24:86. doi: 10.1186/s12864-023-09186-8 (PMC9951484; doi:10.1186/s12864-023-09186-8)
Supplement: Supplementary file 3 — Additional file 3. Figure S3. Motif analysis of differentially expressed genes in the fetal brain after PS. [file 12864_2023_9186_MOESM3_ESM.pdf]

**A**

Up-DEGs_Motifs

| Rank | Motif | Name | P-value | log P-pvalue | q-value (Benjamini) | # Target Sequences with Motif | % of Targets Sequences with Motif | # Background Sequences with Motif | % of Background Sequences with Motif |
|---|---|---|---|---|---|---|---|---|---|
| 1 | 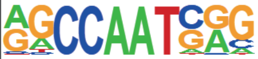 | NFY(CCAAT)/Promoter/Homer | 1e-7 | -1.831e+01 | 0.0000 | 98.0 | 44.14% | 6825.5 | 26.42% |
| 2 | 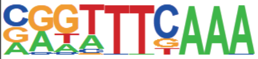 | CHR(?)/Hela-CellCycle-Expression/Homer | 1e-6 | -1.505e+01 | 0.0001 | 36.0 | 16.22% | 1644.9 | 6.37% |
| 3 | 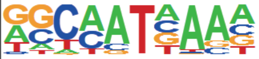 | Hoxa9(Homeobox)/ChickenMSG-Hoxa9.Flag-ChIP-Seq(GSE86088)/Homer | 1e-3 | -7.348e+00 | 0.0945 | 89.0 | 40.09% | 7672.9 | 29.70% |
| 4 | 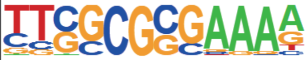 | E2F(E2F)/Hela-CellCycle-Expression/Homer | 1e-3 | -7.310e+00 | 0.0945 | 17.0 | 7.66% | 799.7 | 3.10% |
| 5 | 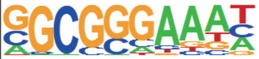 | E2F4(E2F)/K562-E2F4-ChIP-Seq(GSE31477)/Homer | 1e-2 | -6.850e+00 | 0.0945 | 77.0 | 34.68% | 6505.1 | 25.18% |
| 6 | 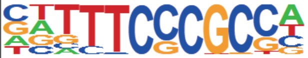 | E2F7(E2F)/Hela-E2F7-ChIP-Seq(GSE32673)/Homer | 1e-2 | -6.420e+00 | 0.1195 | 28.0 | 12.61% | 1782.5 | 6.90% |
| 7 | 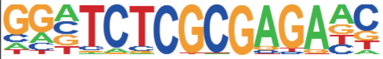 | ZBTB33(Zf)/GM12878-ZBTB33-ChIP-Seq(GSE32465)/Homer | 1e-2 | -5.876e+00 | 0.1763 | 18.0 | 8.11% | 997.2 | 3.86% |

**B**

Down-DEGs_Motifs

| Rank | Motif | Name | P-value | log P-pvalue | q-value (Benjamini) | # Target Sequences with Motif | % of Targets Sequences with Motif | # Background Sequences with Motif | % of Background Sequences with Motif |
|---|---|---|---|---|---|---|---|---|---|
| 1 | 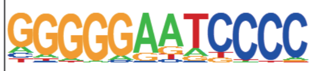 | NFkB-p50,p52(RHD)/Monocyte-p50-ChIP-Chip(Schreiber_et_al.)/Homer | 1e-2 | -5.527e+00 | 1.0000 | 17.0 | 5.70% | 786.2 | 2.72% |
| 2 | 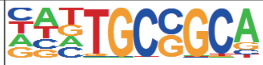 | Zfp57(Zf)/H1-ZFP57.HA-ChIP-Seq(GSE115387)/Homer | 1e-2 | -4.996e+00 | 1.0000 | 57.0 | 19.13% | 3983.4 | 13.80% |
| 3 | 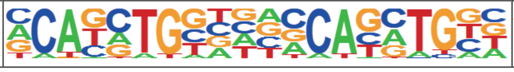 | Twist(bHLH)/HMLE-TWIST1-ChIP-Seq(Chang_et_al)/Homer | 1e-2 | -4.918e+00 | 1.0000 | 11.0 | 3.69% | 442.8 | 1.53% |

**Figure S3: Motif analysis of differentially expressed genes in the fetal brain after PS.** (**A-B**) The motif analysis results of (**A**) up- and (**B**) down-regulated genes. *P*adj < 0.05.
