# Supplementary material for: Transcriptomic profiling of the developing brain revealed cell-type and brain-region specificity in a mouse model of prenatal stress
Source: BMC Genomics. 2023 Feb 24;24:86. doi: 10.1186/s12864-023-09186-8 (PMC9951484; doi:10.1186/s12864-023-09186-8)
Supplement: Supplementary file 4 — Additional file 4. Figure S4. Cell deconvolution analysis. [file 12864_2023_9186_MOESM4_ESM.pdf]

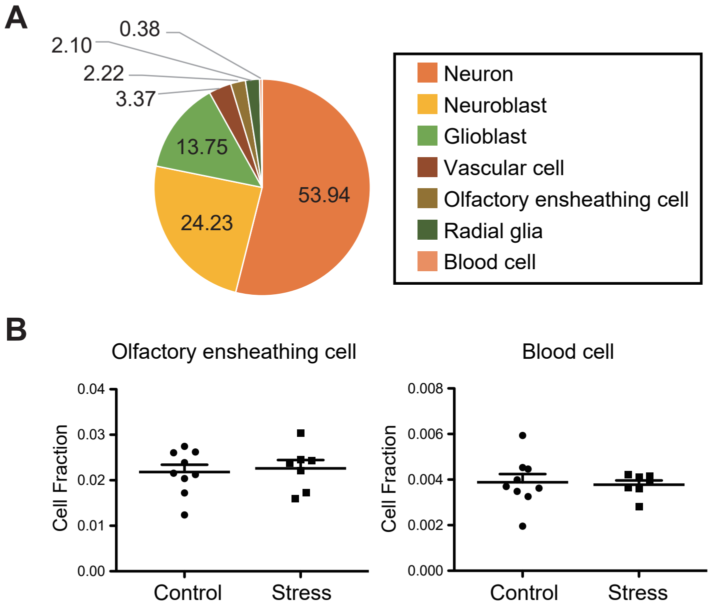

Figure S4: Cell deconvolution analysis. (**A**) Pie chart shows cell fraction of the fetal brain calculated by cell deconvolution analysis of the current bulk RNA-seq using published scRNA-seq. (**B**) Bar graphs show cell fractions (olfactory ensheathing cell and blood cell) in control and stressed brains. N=9 control, 7 stress. Mean ± SEM. Unpaired t-test.
