# Supplementary material for: Transcriptomic profiling of the developing brain revealed cell-type and brain-region specificity in a mouse model of prenatal stress
Source: BMC Genomics. 2023 Feb 24;24:86. doi: 10.1186/s12864-023-09186-8 (PMC9951484; doi:10.1186/s12864-023-09186-8)
Supplement: Supplementary file 5 — Additional file 5. Figure S5. Schematic diagram shows sagittal sections picked from lateral to midline for Figure 4. [file 12864_2023_9186_MOESM5_ESM.pdf]

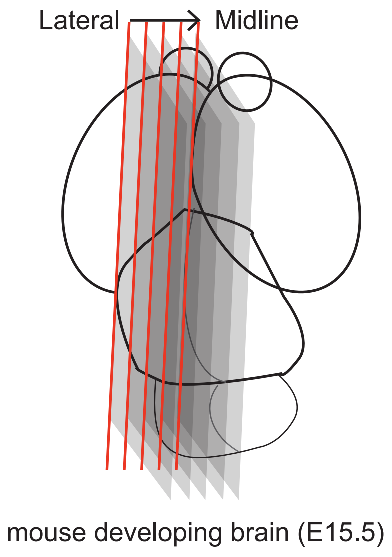

Figure S5: Schematic diagram shows sagittal sections picked from lateral to midline for Figure 4.
